# The quality and reliability of short videos about depression on TikTok (Douyin): a cross-sectional study

**DOI:** 10.1038/s41598-026-45237-2

**Published:** 2026-03-21

**Authors:** Yanbin Lin, Hongli Tao, Lina Wang, Guoying Sun, Shanshan Zhao, Bang An

**Affiliations:** Department of Psychiatry, Xianyang Central Hospital, No. 78, East Renmin Road, Xianyang, 712000 China

**Keywords:** Depression, TikTok, Douyin, Short videos, Social media, Video quality, Patient education, Diseases, Health care, Psychology, Psychology

## Abstract

Depression is one of the most prevalent mental health disorders worldwide. Despite its significant impact on patients’ well-being, its etiology remains unclear, and diagnosis is often challenging. Currently, effective depression management requires long-term patient engagement, which includes consistent self-care, adherence to treatment plans, and ongoing mental health literacy. However, the quality of depression-related videos on short-video platforms varies widely, and some may contain misinformation that patients struggle to identify. This study aims to assess the information quality of depression-related short videos on TikTok (Douyin), the largest short-video platform in China. Based on the composite ranking, the top 200 depression-related videos on TikTok (Douyin) were rigorously screened by two independent psychiatrists according to predefined inclusion/exclusion criteria. Videos were categorized by publisher type, and detailed engagement metrics (likes, shares, comments, favorites) were recorded. Video quality was assessed using the Global Quality Scale (GQS), JAMA Benchmark Criteria, and the modified DISCERN tool (mDISCERN). Content analysis covered six key domains: definition, symptoms, risk factors, diagnosis, treatment, and outcomes. Finally, correlation analysis was conducted to evaluate the relationship between video quality and engagement metrics. The study ultimately included 121 depression-related videos, the majority of which (62%) were published by healthcare professionals, including psychiatrists (39.7%) and psychologists (13.2%). Overall, video quality was low, with median scores of 2 (IQR: 1–3) for both GQS and JAMA, and 0 (IQR: 0–1) for mDISCERN. In contrast, videos authored by psychiatrists provided more comprehensive coverage of depression-related content and demonstrated superior reliability and quality, with median scores of 3 (IQR: 2–3) for DISCERN, 2.5 (IQR: 2–3) for GQS, and 1 (IQR: 1–2) for JAMA. Notably, psychiatrist-produced videos also achieved higher public engagement metrics. Correlation analysis revealed a significant positive association between video quality scores and public engagement. Overall, the quality of depression-related video content on TikTok (Douyin) remains suboptimal and requires significant improvement. There is a pressing need for more high-quality, evidence-based depression-related videos to facilitate long-term and effective self-management among individuals with depression. Simultaneously, such efforts would help improve public awareness and accurate understanding of depression.

**Supplementary Information:**

The online version contains supplementary material available at 10.1038/s41598-026-45237-2.

## Introduction

Depression is a prevalent mental disorder characterized by persistent low mood, diminished interest, impaired cognitive function, and physical manifestations including sleep disturbances and appetite changes. As a major global public health concern, depression affects approximately 3.8% of the world’s population according to recent WHO statistics. The disorder demonstrates higher prevalence among adults, with women affected at 1.5-2 times the rate of men^1^. Notably, incidence rates among adolescents and young adults have risen dramatically by 40% over the past decade. Beyond reducing quality of life, depression significantly elevates suicide risk, contributing to nearly 700,000 annual deaths and imposing a global economic burden exceeding 1 trillion per year^2^.

While depression often follows a recurrent course and can be challenging to cure completely, long-term management strategies offer substantial benefits^3^. Preventive and therapeutic interventions including pharmacotherapy, psychotherapy, lifestyle modifications, and mental health skills training can effectively mitigate both initial onset and recurrence risks^4,5^. Nevertheless, most patients lack adequate understanding of depression and standardized treatment protocols, frequently delaying diagnosis and treatment initiation. Medication non-adherence exemplifies this challenge, escalating from 28% in the first month to 64% by the sixth month of treatment. Such nonadherence not only compromises therapeutic outcomes but may also precipitate pharmacogenetic adverse effects, ultimately contributing to treatment-resistant recurrence^6^.

The digital revolution has transformed mental health information dissemination, with TikTok-style short video platforms emerging as dominant health communication channels^7^. In China, TikTok (known as Douyin) is the most dominant short-form video platform, with over 700 million monthly active users, making it a primary source of health information for many, particularly among younger demographics. Content spans various chronic and mental health conditions, including diabetes, hypertension, schizophrenia, and depression. These platforms leverage fragmented content delivery, high interactivity, and algorithmic personalization to engage audiences effectively^8,9^. While offering unprecedented access to depression-related information spanning etiology, symptomatology, diagnosis, and treatment options, this new paradigm presents a dual-edged sword. The engaging, digestible format facilitates mental health literacy^10^, yet variable content quality raises concerns about potential misinformation^11–13^. Specifically concerning clinical depression, misinformation on social media can lead to delayed help-seeking, increased stigma, inappropriate self-management attempts, and exacerbate outcomes for individuals with difficult-to-treat or treatment-resistant depression^14^.

Despite the growing influence of short video platforms in health education, systematic evaluation of depression-related content remains lacking. Our study addresses this critical gap by assessing information quality on TikTok (Douyin). Through rigorous evaluation, we aimed to establish a snapshot of current content quality and identify prevalent gaps, which can inform future efforts to improve health communication on such platforms. These efforts seek to enhance public depression literacy, support early recognition, and empower patients through improved health literacy and self-management skills, ultimately reducing recurrence risk and improving quality of life.

**Fig. 1:**
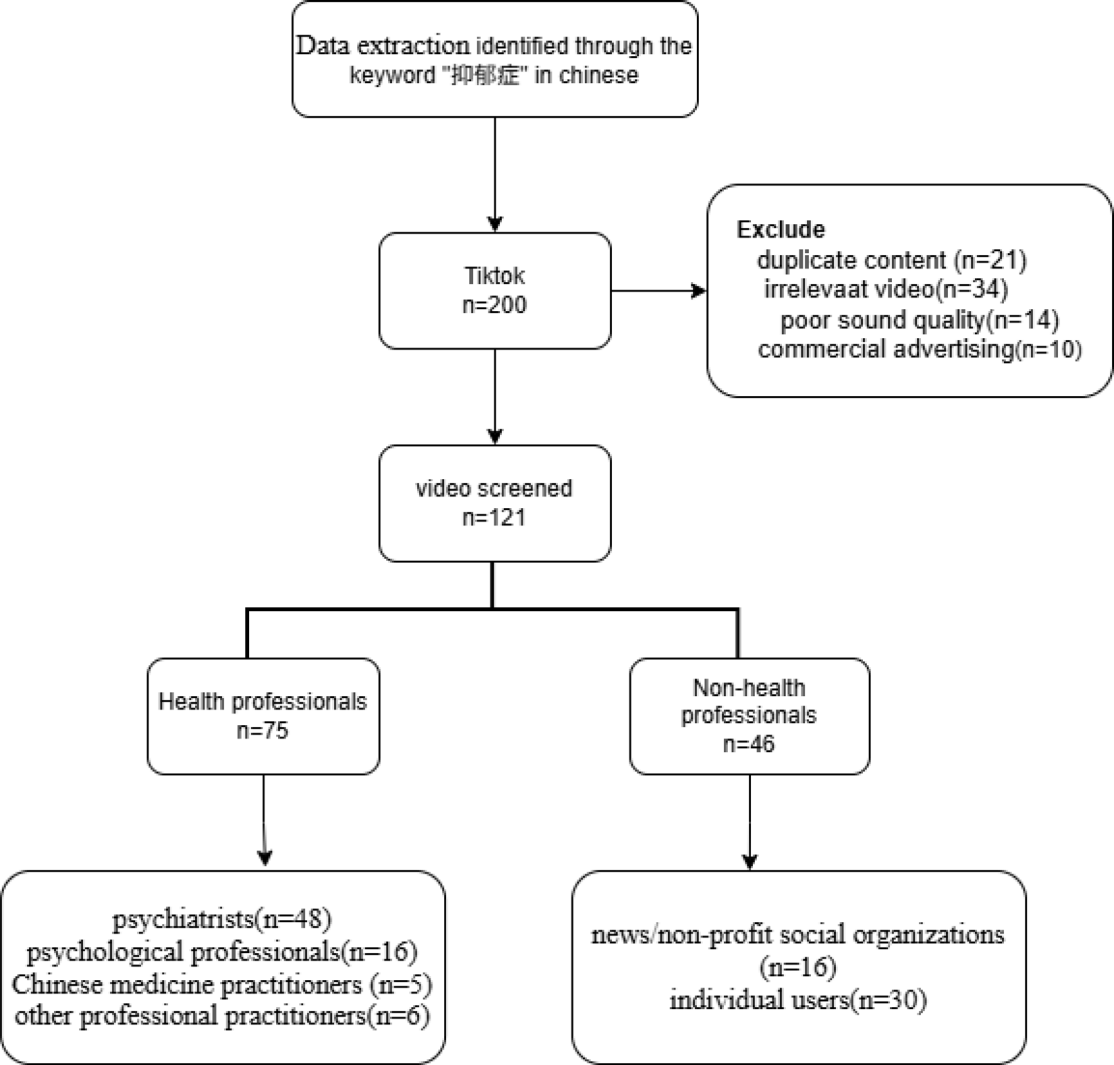
The flow chart of this study.

## Materials and methods

### Search strategy and data extraction

The purpose of this study was to collect video data related to depression from TikTok (Douyin). To ensure the comprehensiveness and representativeness of the data, the Chinese term “抑郁症” was used as the search keyword. Before conducting the video searches, to minimize bias from personalized recommendations, we performed the following steps on our smartphones: (1) uninstalled and reinstalled the TikTok (Douyin) app; (2) cleared all application cache and data; (3) created a new user account without any profile information or viewing history; and (4) conducted all searches in ‘incognito’ or private browsing mode where available. All searches were performed from a single location (Xianyang, China) using devices running iOS/Android OS, between July 20–30, 2025.

Initially, the top 200 videos were collected sequentially based on TikTok’s default composite ranking (a platform-generated ranking that considers factors like recent popularity and engagement, though it may still be influenced by underlying algorithmic curation). The inclusion and exclusion criteria were as follows:

Inclusion criteria: Videos primarily discussing depression (major depressive disorder).

Exclusion criteria: (a) Duplicate content (identical videos or re-uploads by the same or different accounts); (b) Content not directly related to clinical depression (e.g., general sadness, economic depression); (c) Videos with no audio or poor audio quality hindering comprehension; (d) Videos primarily used for commercial advertising of products or services.

After screening, 121 videos were finally obtained for inclusion in this study.

The following basic information was recorded in detail during the data extraction process, including the identity of the publisher, posting time, duration, number of likes, favorites, and shares. Video publishers were categorized into two main groups: health professionals and non-health professionals. These categorizations were based on the publisher’s account name, self-description, and platform verification status (e.g., “professional certification” badge).

The health professionals group included: psychiatrists, psychologists, Chinese medicine practitioners, and other healthcare practitioners (e.g., general practitioners, nurses). The non-health professionals group included: news agencies/non-profit social organizations and individual users/science bloggers.

Two independent reviewers (Tao HL and Wang LN), both psychiatrists from Xianyang Central Hospital, conducted the initial search, screening, and data extraction. A third researcher (Lin YB) was available to adjudicate disagreements. The quality of the basic information and content of the videos was recorded and assessed by the same two independent reviewers. Any disagreements were resolved by consultation with the third researcher. We ensured that no videos were downloaded, retweeted, liked, or commented on during the evaluation process to prevent any interference with the data.

### Assessment tools

The quality and reliability of the videos were evaluated using three commonly applied instruments: the modified DISCERN (mDISCERN), the Journal of the American Medical Association (JAMA) benchmark criteria, and the Global Quality Scale (GQS)^15^.

The modified DISCERN (mDISCERN) tool was adapted from the original DISCERN instrument developed to evaluate the quality of written consumer health information^16^. The tool assesses five aspects of information reliability, including clarity of aims, relevance of information, transparency of sources (attribution), balance or unbiased presentation (fairness), and overall reliability. Each item is rated as “yes” (1 point) or “no” (0 points), resulting in a total score ranging from 0 to 5, with higher scores indicating greater reliability. The DISCERN instrument has been widely applied in studies evaluating online health information and has demonstrated good reliability and validity^16^. Previous studies assessing social media health content have also reported good inter-rater reliability for the mDISCERN, with Cohen’s kappa values often exceeding 0.70^17^.

The JAMA benchmark criteria evaluate the credibility and transparency of online health information across four domains: authorship, currency, disclosure of conflicts of interest, and attribution (references and sources)^18^. Each criterion is scored as 1 (criterion met) or 0 (not met), yielding a total score ranging from 0 to 4. The JAMA benchmarks were originally proposed to assess the quality and accountability of medical information on the internet and have been widely used in studies evaluating online health resources^18^.

The Global Quality Scale (GQS) is a 5-point Likert scale (1 = very poor quality, 5 = very high quality) used to assess the overall educational quality of health-related videos^19^. The evaluation considers factors such as the flow of information, comprehensiveness, clarity, and usefulness for viewers. The GQS has been frequently used in studies assessing medical information on video-sharing platforms and has demonstrated excellent inter-rater reliability, with high intraclass correlation coefficients (ICCs) reported in previous analyses^20^.

In addition, we used a content completeness scoring rubric adapted from Goobie et al.^21^.and He et al.^22^.to assess video content in six domains relevant to depression: definition, symptoms, risk factors, diagnosis/tests, treatment/management, and outcomes. Each domain was scored as: 0 = no content mentioned; 1 = content mentioned but minimal or superficial; 2 = substantial, detailed content provided. To ensure consistency, a detailed scoring guide with examples for each level was provided to raters.

### Rater procedure and reliability

To ensure methodological rigor, an independent double-rating process with adjudication was implemented. The two primary raters (Tao HL and Wang LN) received standardized training on the assessment tools and scoring rubrics prior to the study. They independently evaluated all videos. Inter-rater reliability was calculated using Cohen’s kappa (κ) for categorical items (JAMA, content completeness) and Intraclass Correlation Coefficient (ICC) for continuous/ordinal scales (GQS, mDISCERN total score). Any discrepancies between the two raters were discussed, and if consensus was not reached, a third researcher (Lin YB) made the final decision.

### Statistical analysis

Statistical analysis was performed using IBM SPSS Statistics (version 26.0) and Origin 2024. The normality of continuous variables was assessed using the Shapiro-Wilk test. For comparisons of video characteristics and quality scores across multiple publisher categories (e.g., psychiatrists, psychologists, etc.), the Kruskal-Wallis H test was used as the non-parametric alternative to one-way ANOVA, followed by post-hoc Dunn’s tests with Bonferroni correction for pairwise comparisons where overall significance was found. For comparisons between two independent groups (health vs. non-health professionals), the Mann-Whitney U test was used for non-normally distributed data. Spearman’s rank correlation coefficient (ρ) was used to assess correlations between ordinal/non-normal variables (e.g., quality scores and engagement metrics). Continuous variables that did not follow a normal distribution were reported as median (interquartile range, IQR). Categorical variables were expressed as frequencies and percentages. Inter-rater reliability between the two reviewers was assessed using Cohen’s kappa coefficient for the mDISCERN and JAMA scores and the intraclass correlation coefficient (ICC) for the GQS scores. P-values < 0.05 were considered statistically significant.

**Table 1:** Video characteristics. GQS: Global Quality Score; IQR: interquartile range.

Characteristics	N = 121
Video source	
Health professionals	75 (62%)
Psychiatrist, No. (%)	48 (39.7%)
Psychologist, No. (%)	16 (13.2%)
Chinese medicine, No. (%)	5 (4.1%)
Other specialties, No. (%)	6 (5.0%)
Non-health professionals	46 (38%)
News agencies/Nonprofit organizations, No. (%)	16 (13.2%)
Science blogger, No. (%)	30 (24.8%)
Video duration (seconds), median, IQR	51 (35.2–66.5)
Number of likes, median, IQR	281 (44.5-2126.5)
Number of shares, median, IQR	71 (9-930)
Number of collections median, (IQR)	83 (10.5-775.5)
Number of comments median, (IQR)	47 (9-310)
JAMA score median, (IQR)	0 (0–1)
GQS scores median, (IQR)	2 (1–3)
mDISCERN scores median, (IQR)	2 (1–3)

## Results

**Fig. 2:**
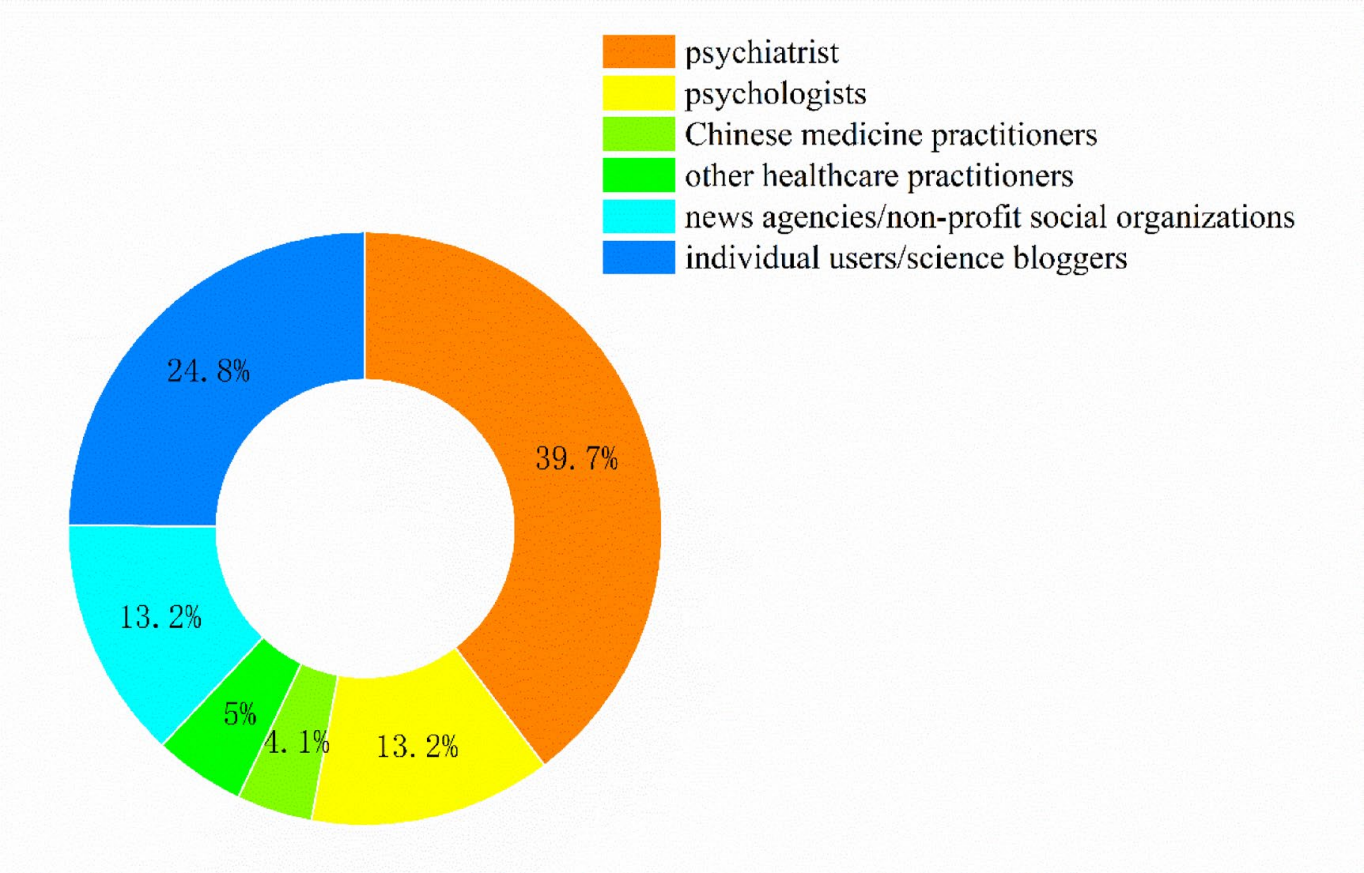
Video source distribution.

### Characteristics of included videos

The top 200 short videos from TikTok’s (Douyin’s) overall ranking were screened according to the study’s inclusion and exclusion criteria, and a final sample of 121 videos was obtained; the detailed screening process is shown in Fig. 1. Table 1 shows the characteristics of the included videos. Based on the identity of the video’s publisher, videos were categorized into health professionals (75, 62.0%) and non-health professionals (46, 38.0%). Health professionals were further categorized into psychiatrists (48, 39.7%), psychologists (16, 13.2%), Chinese medicine practitioners (5, 4.1%), and other healthcare-related personnel (6, 5.0%). Non-health professionals mainly included news agencies/non-profit organizations (16, 13.2%) as well as science bloggers/individual users (30, 24.8%) (Fig. 2). The median video duration was 51 s (IQR: 35.2–66.5). The median number of likes, collections, comments, and shares were 281 (IQR: 44.5–2126.5), 83 (IQR: 10.5–775.5), 47 (IQR: 9–310), and 71 (IQR: 9–930), respectively (Table 1). Video quality was assessed using three tools: GQS, mDISCERN, and JAMA, with median scores of 2 (IQR: 1–3), 2 (IQR: 1–3), and 0 (IQR: 0–1), respectively (Table 1).

**Table 2:** Completeness of video content.

Video content	Definition n (%)	Signs/symptoms n (%)	Diagnosis/Tests n (%)	Treatment/Management n (%)	Risk factors n (%)	Outcomes, n (%)
No content (0 points)	61(50.4)	11(9.1)	83(68.6)	57(47.1)	36(29.8)	76(62.8)
Little content (1 point)	53(43.8)	88(72.7)	34(28.1)	55(45.5)	73(60.3)	42(34.7)
Substantial content (2 points)	7(5.8)	22 (18.2)	4 (3.3)	9 (7.4)	12 (9.9)	3 (2.5)

### Short video content analysis

The completeness of the content of each video was assessed based on six main domains: definition, symptoms, risk factors, diagnosis/tests, treatment/management, and outcomes (Table 2). Few videos provided comprehensive information related to depression. Specifically, 68.6% of the videos did not mention depression-related diagnostic tests, 62.8% did not mention disease outcomes, and 47.1% did not mention treatments. The definition of depression was not mentioned in 50.4% of the videos. In contrast, symptoms and risk factors were mentioned more frequently, covered in 90.9% and 70.2% of videos, respectively. However, only a small proportion of videos provided substantial details on these topics: definition (5.8%), symptoms (18.2%), risk factors (9.9%), diagnosis/tests (3.3%), treatment (7.4%), and outcomes (2.5%).

**Table 3:** Comparison of depression-related video characteristics according to video source.

Video features	Psychiatrists (n = 48)	Psychologist (n = 16)	Chinese medicine (n = 5)	Other specialties (n = 6)	News agencies/Nonprofit (n = 16)	Science blogger/Individual (n = 30)	P
Video duration(seconds), median (IQR)	50.5(32.25-57)	52(31.25-64)	58(43–84)	34(72.5–21.5)	57(31-88.75)	48(36-124.75)	0.409
Number of likes, median (IQR)	423.5(61.25-2566.75)	377.5(132.25–22690)	24(19-58.5)	49.5(17.25-312.25)	194(8.25-1140.50)	175(16.75–2093)	0.010
Number of shares, median (IQR)	119(17.25–769.5)	147.5(50-7119.25)	41(7–59)	12(1-33.5)	39(1-298.25)	29.5(3-114.2)	0.018
Number of collections, median (IQR)	110(14.25–730.5)	95.5(28.5-5024.25)	19(8–60)	12.5(4.5-153.25)	34.5(1-354.5)	33.5(3.75-1010.5)	0.133
Number of comment, median (IQR)	60.5(15.25-297.75)	78(20.25–3081)	22(7.5–49)	11(3-31.5)	33(2-129.75)	45(3.75–166.5)	0.046
JAMA score, median (IQR)	1(1–2)	1(1–4)	1(0-1.5)	0(0–1)	1(0–1)	0(0–1)	0.002
GQS scores, median (IQR)	2.5(2–3)	2(2-3.75)	2(1.5–2.5)	1.5(1-2.25)	1.5(1–2)	1(1–2)	< 0.001
mDISCERN scores, median (IQR)	3(2–3)	3(2–4)	2(1.5-3)	2(0.75-2)	1(0-2.75)	1.5(0–3)	0.001

IQR: interquartile range; GQS: Global Quality Score; mDISCERN: modified DISCERN.

### Comparison of videos from different sources

**Fig. 3:**
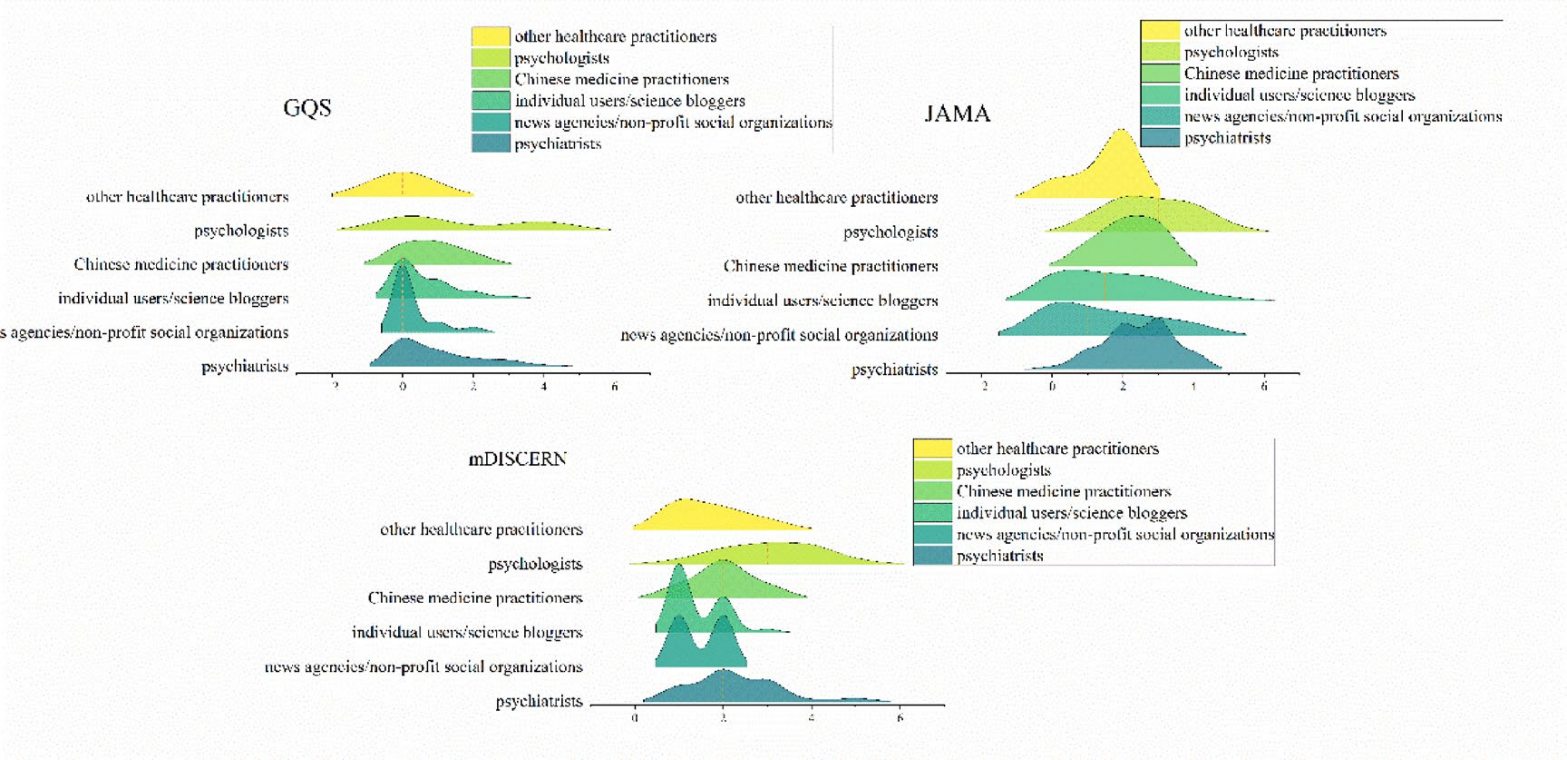
Comparison between Global Quality Scores (GQS), JAMA and mDISCERN scores across video sources.

As shown in Table 3, videos were compared across different publisher categories. Kruskal-Wallis tests revealed significant differences among groups for several engagement and quality metrics. Psychiatrist-produced videos received the highest median number of likes, while psychologist-produced videos received the highest median number of shares and comments. No significant differences were found among groups for the number of collections and video duration (*p* > 0.05). In terms of quality, videos posted by psychiatrists received significantly higher GQS (*p* < 0.001), JAMA (*p* = 0.002), and mDISCERN (*p* = 0.001) scores compared to other groups. Post-hoc pairwise comparisons indicated that psychiatrists’ videos had significantly higher quality scores than those from news agencies, individual users, and “other specialties” in most comparisons (Fig. 3).

### Correlation analysis

**Fig. 4:**
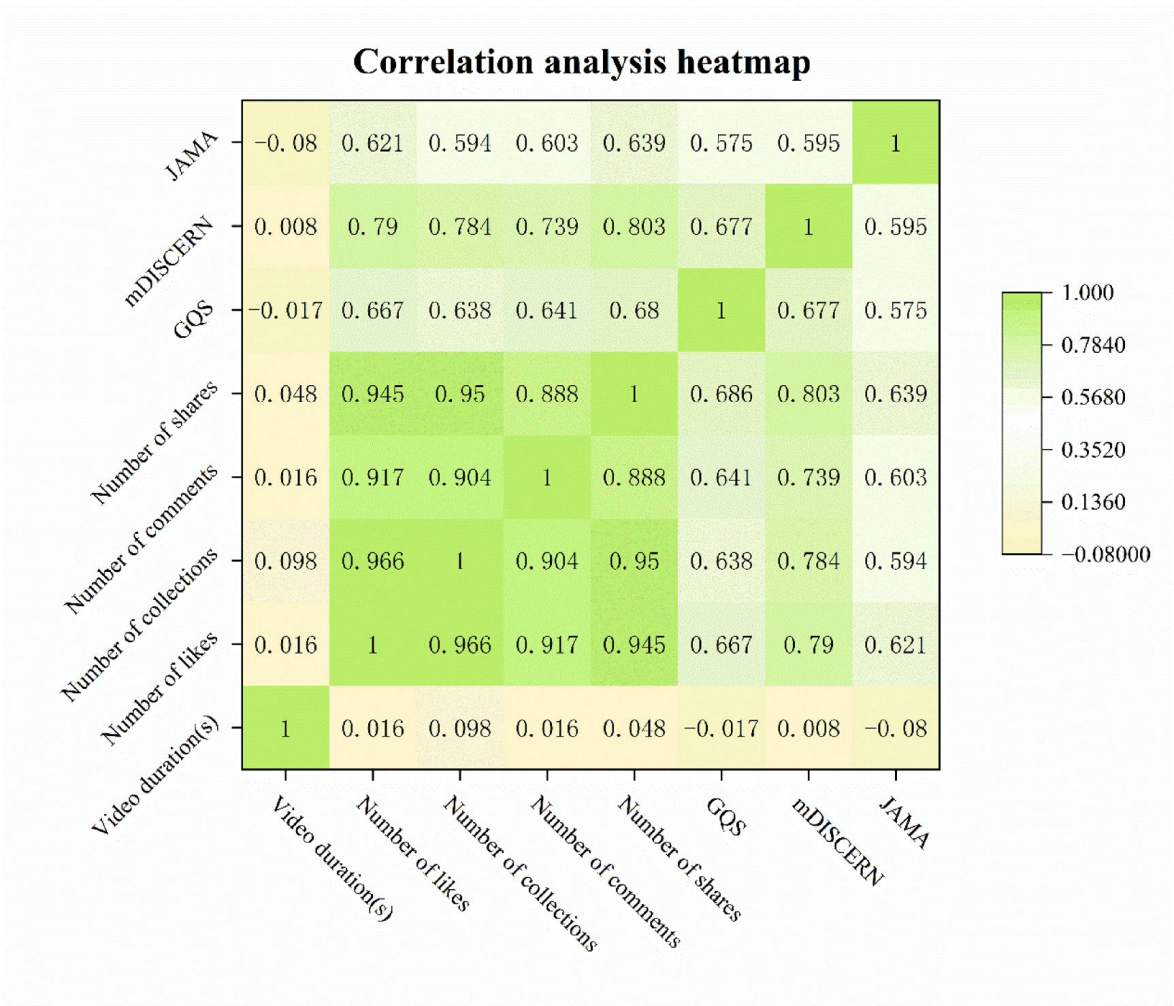
Heatmap of correlations between quality scores and engagement metrics.

Spearman correlation analysis was used to examine the relationships between quality scores and engagement metrics (Fig. 4). Significant positive correlations were found between engagement metrics and content quality scores. JAMA scores were significantly correlated with the number of likes (ρ = 0.25, *p* = 0.006), collections (ρ = 0.22, *p* = 0.016), shares (ρ = 0.26, *p* = 0.004), as well as with mDISCERN (ρ = 0.45, *p* < 0.001) and GQS scores (ρ = 0.40, *p* < 0.001). Similarly, mDISCERN scores were significantly correlated with likes (ρ = 0.30, *p* < 0.001), collections (ρ = 0.28, *p* = 0.002), shares (ρ = 0.31, *p* < 0.001), and GQS scores (ρ = 0.65, *p* < 0.001). GQS scores were also correlated with likes (ρ = 0.36, *p* < 0.001), comments (ρ = 0.21, *p* = 0.022), collections (ρ = 0.34, *p* < 0.001), and shares (ρ = 0.37, *p* < 0.001). Notably, video duration was not significantly correlated with JAMA scores (ρ = 0.08, *p* = 0.383), GQS scores (ρ = -0.01, *p* = 0.894), or mDISCERN scores (ρ = -0.01, *p* = 0.933).

## Discussion

TikTok (Douyin), TikTok (Douyin), one of the world’s fastest-growing short-form video platforms with over 1 billion users, has become an increasingly influential channel for health information dissemination^7,23^. However, concerns regarding the quality and reliability of health-related content on this platform have been widely reported^24–29^. In this study, we systematically evaluated depression-related videos on TikTok (Douyin) and identified several key findings. Overall, the informational quality and reliability of the videos were generally low, with substantial variation depending on the creator’s professional background. Videos produced by psychiatrists and other healthcare professionals demonstrated significantly higher quality and reliability compared with those created by non-health professionals. In addition, many videos lacked comprehensive information regarding diagnostic criteria and long-term outcomes of depression.

These findings are consistent with previous studies evaluating health-related content on TikTok and other social media platforms. Research on schizophrenia and diabetes-related videos has similarly reported variable information quality and limited adherence to evidence-based standards^24,25^. In the present study, fewer than 40% of videos were created by psychiatrists, and a considerable proportion of content originated from non-health professionals. Such creators often produced videos lacking clinical accuracy or sufficient depth, potentially limiting the platform’s usefulness as a reliable source of patient education.

Our analysis also revealed notable imbalances in informational content. More than half of the videos did not address essential aspects of depression, including its definition, diagnostic criteria, or long-term outcomes, and instead focused mainly on symptoms and risk factors. Similar patterns have been observed in previous studies of short-form health videos, where simplified and symptom-focused information tends to dominate due to time constraints and audience engagement considerations^8,15,22^. When stratified by creator background, healthcare professionals—particularly psychiatrists—provided more comprehensive coverage across multiple domains, including definitions, symptoms, risk factors, diagnosis, and prognosis. In contrast, non-specialist creators more frequently emphasized experiential or symptom-based content rather than clinically structured explanations.

Regarding audience engagement, videos posted by psychiatrists received significantly higher numbers of likes, comments, and shares than those produced by non-health professionals, although video length and number of collections did not differ significantly between groups. Psychologists also demonstrated relatively high levels of content quality and audience engagement, second only to psychiatrists. This may reflect the central role of psychotherapy in the management of depression. Previous studies have similarly indicated that health-related content produced by medical professionals generally demonstrates higher informational quality than that produced by non-professionals^30^.

Correlation analysis revealed significant positive associations between video engagement metrics (likes, shares, comments, and collections) and content quality scores. However, this association should be interpreted cautiously. Engagement metrics on social media platforms may be influenced by multiple factors, including creator popularity, follower base, recommendation algorithms, posting time, and production quality. Therefore, the observed correlation does not necessarily indicate that viewers are able to accurately judge the scientific quality of health information. Similar inconsistencies between engagement and information quality have also been reported in previous TikTok-based studies^24,25,27^.

The generally low quality of depression-related videos identified in this study raises concerns about the potential impact of misinformation. Depression is a chronic psychiatric disorder with a high risk of recurrence and often requires long-term management^3,4,31^. Individuals seeking health information online, particularly those experiencing depressive symptoms, may encounter incomplete or inaccurate information that could lead to misunderstanding of the disorder or delays in seeking professional treatment^13,30^. This concern may be especially relevant for individuals with treatment-resistant depression, who already face significant challenges in obtaining effective care^14^.

Several limitations should be acknowledged. First, the sample was based on TikTok’s (Douyin’s) “default composite ranking” at a single time point. Because this ranking is algorithm-driven and influenced by user behavior, geographic location, and temporal trends, the selected videos may not fully represent the broader range of available content. Second, the study focused exclusively on TikTok (Douyin) within the Chinese context, which may limit the generalizability of the findings to other platforms or cultural settings. Third, the dynamic nature of recommendation algorithms on short-video platforms means that video visibility and dissemination patterns may change over time, which cannot be captured in a cross-sectional study. Fourth, although standardized assessment tools were used, some degree of subjectivity remains in the evaluation process, and the simplified content completeness framework may not capture all dimensions of educational quality. Finally, the cross-sectional design limits causal interpretation of the observed associations.

Future studies should address these limitations by conducting repeated sampling across different time points to reduce algorithmic bias, including multiple platforms and languages, and incorporating additional indicators of creator influence such as follower counts and account verification status. More detailed and validated evaluation frameworks may further improve the assessment of online health information quality. Longitudinal or experimental studies may also help clarify the relationship between content quality, user engagement, and the dissemination of health information on short-video platforms.

## Conclusion

This study evaluated the quality of information in depression-related videos on TikTok (Douyin). The results indicate that the overall quality of such videos is low, with significant gaps in comprehensive content, particularly regarding diagnosis, treatment, and outcomes. Video quality varied significantly by source, with videos from psychiatrists and psychologists generally scoring higher on quality metrics. Based on our assessment, viewers seeking depression information may benefit from prioritizing content created by verified mental health professionals. However, the observed positive association between quality scores and engagement should not be interpreted as causal evidence of audience discrimination. There is a clear need for more high-quality, evidence-based content on depression. Platform developers, health professionals, and public health authorities could collaborate to improve content standards and visibility of reliable information, potentially enhancing the platform’s role as a tool for public mental health literacy.

## Supplementary Information

Below is the link to the electronic supplementary material.

Supplementary Material 1

## Data Availability

The original data used in this study were obtained from TikTok (Douyin) (https://www.douyin.com/) and are publicly available on the platform. The analyzed datasets generated during the study are available from the corresponding author upon reasonable request.
