# Supplementary material for: The quality and reliability of short videos about depression on TikTok (Douyin): a cross-sectional study
Source: Sci Rep. 2026 Mar 21;16:14372. doi: 10.1038/s41598-026-45237-2 (PMC13144367; doi:10.1038/s41598-026-45237-2)
Supplement: Supplementary file 1 — Supplementary Material 1 [file 41598_2026_45237_MOESM1_ESM.pdf]

Supplementary Table 2: Global Quality Score (GQS) Scale (Scoring from 1 to 5)

GQS Definition	Score
Lacks clarity and coherence, with significant information gaps, offering minimal value to patients	1
Substandard in clarity and structure, includes some details but omits critical topics, limiting its utility for patients	2
Moderate quality, with key information sufficiently covered	3
Highly informative with smooth progression, covers most relevant information, valuable for patients	4
Outstanding in both content and delivery, exceptionally beneficial for patients	5

Table S1. The Journal of the American Medical Association (JAMA) benchmark criteria.

Score*	Score component	
1 score	Authorship	Author and contributor credentials and their affiliations should be provided.
1 score	Attribution	Clearly lists all copyright information and states references and sources for content.
1 score	Currency	Initial date of posted content and subsequent updates to content should be provided.
1 score	Disclosure	Conflicts of interest, funding, sponsorship, advertising, support, and video ownership should be fully disclosed.

*The criteria of each aspect were scored separately, and 1 point for each criterion with a total score of 4 points.

Supplementary Table 1: Modified DISCERN quality assessment criteria for evaluating video reliability. (Award 1 point for a 'yes' response, 0 points for a 'no' response)

**Reliability Score**
1. Does the video convey information in a clear, succinct, and easily comprehensible manner?
2. Are references to credible sources provided?
3. Is the material presented impartially and without favoritism?
4. Are supplementary content resources indicated for further patient exploration?
5. Are any uncertain aspects of the content acknowledged?
